# Foliar Application of Different Vegetal-Derived Protein Hydrolysates Distinctively Modulates Tomato Root Development and Metabolism

**DOI:** 10.3390/plants10020326

**Published:** 2021-02-08

**Authors:** Angela Valentina Ceccarelli, Begoña Miras-Moreno, Valentina Buffagni, Biancamaria Senizza, Youry Pii, Mariateresa Cardarelli, Youssef Rouphael, Giuseppe Colla, Luigi Lucini

**Affiliations:** 1 Department of Agriculture and Forest Sciences, University of Tuscia, 01100 Viterbo, Italy; avceccarelli@unitus.it; 2 Department for Sustainable Food Process, Research Centre for Nutrigenomics and Proteomics, Università Cattolica del Sacro Cuore, 29122 Piacenza, Italy; mariabegona.mirasmoreno@unicatt.it (B.M.-M.); valentina.buffagni@unicatt.it (V.B.); biancamaria.senizza@unicatt.it (B.S.); luigi.lucini@unicatt.it (L.L.); 3 Faculty of Science and Technology, Free University of Bozen, 39100 Bolzano, Italy; youry.pii@unibz.it; 4 Consiglio per la Ricerca in Agricoltura e L’analisi Dell’economia Agraria, Centro di Ricerca Orticoltura e Florovivaismo, 84098 Pontecagnano Faiano, Italy; mteresa.cardarelli@crea.gov.it; 5 Department of Agricultural Sciences, University of Naples Federico II, 80055 Portici, Italy

**Keywords:** biostimulants, metabolomics, hormone-like activity, plant bioassay, *Solanum lycoperscum* L.

## Abstract

Despite the scientific evidence supporting their biostimulant activity, the molecular mechanism(s) underlying the activity of protein hydrolysates (PHs) and the specificity among different products are still poorly explored. This work tested five different protein hydrolysates, produced from different plant sources using the same enzymatic approach, for their ability to promote rooting in tomato cuttings following quick dipping. Provided that all the different PHs increased root length (45–93%) and some of them increased root number (37–56%), untargeted metabolomics followed by multivariate statistics and pathway analysis were used to unravel the molecular processes at the basis of the biostimulant activity. Distinct metabolomic signatures could be found in roots following the PHs treatments. In general, PHs shaped the phytohormone profile, modulating the complex interaction between cytokinins and auxins, an interplay playing a pivotal role in root development, and triggered a down accumulation of brassinosteroids. Concerning secondary metabolism, PHs induced the accumulation of aliphatic glucosinolates, alkaloids, and phenylpropanoids, potentially eliciting crop resilience to stress conditions. Here, we confirm that PHs may have a hormone-like activity, and that their application can modulate plant growth, likely interfering with signaling processes. Noteworthy, the heterogenicity of the botanical origin supported the distinctive and peculiar metabolomic responses we observed across the products tested. While supporting their biostimulant activity, these findings suggest that a generalized crop response to PHs cannot be defined and that specific effects are rather to be investigated.

## 1. Introduction

Nowadays, several drawbacks threaten food security, including land degradation and usage, reduced water, and nutrient availability of soils. In this scenario, the improvement of sustainable farming has become a major goal in agriculture, with particular regard to methods that can boost crop productivity and quality and, at the same time, safeguard the environment. In recent years, biostimulants have gained significant interest as innovative “green” products that can foster plant growth and development throughout the crop life cycle in both optimal and sub-optimal growing conditions [1]. Biostimulants were first defined by Kauffman and colleagues [2] as materials which can promote plant growth when applied in low quantities, distinguishing biostimulants from fertilizers, that are used in larger amounts. Nowadays, the European Biostimulants Industry Council (EBIC) refers to biostimulants as any “substance(s) and/or micro-organisms whose function, when applied to plants or the rhizosphere, is to stimulate natural processes to enhance/benefit nutrient uptake, nutrient efficiency, tolerance to abiotic stress, and crop quality”, as set out in the European Regulation 2019/1009 (https://eur-lex.europa.eu/eli/reg/2019/1009/oj (accessed on 5 January 2021)). A promising class of biostimulants is represented by the protein hydrolysates (PHs), a mixture of polypeptides, oligopeptides, and amino acids derived by (partial) hydrolysis of different protein-rich sources [3]. Currently, different PHs are available on the market as derived from the enzymatic hydrolysis of agro-industrial by-products from either animal or vegetal sources [4], of which plant-derived PHs are considered more environmentally-friendly [5] and are widely accepted by farmers due to their efficiency in enhancing crop performances [4]. Indeed, either as foliar or root application, PHs can prompt several alterations in crops’ metabolism and physiology, promoting plant growth and development [6,7], as well as the response to environmental constraints of a wide range of horticultural crops, including different cereal species, lettuce, spinach, and tomato [6,8,9,10,11]. Benefits on crops’ performances induced by PHs directly reflect the molecular reprogramming of both primary and secondary metabolism, which in return improves nutrient use efficiency (NUE) of plants [12,13]. The pivotal role of the root system in nutrient uptake is well established in horticultural crops. Many authors demonstrated how PHs modify root architecture (i.e., root biomass and density, length, and the number of lateral root branching), increasing the overall surface area of the root system, which ensures a better access to nutrient sources in the soil [14,15,16].

Despite the growing evidence on the benefits arising from PH biostimulants, the exact mechanism of PHs is still poorly understood. Some pieces of evidence suggest a hormone-like activity, presumably related to bioactive compounds mimicking the mode of action of peptide phytohormones [4]. In turn, hormones signal transduction pathways modulate plant growth and development, and shape secondary metabolism biosynthetic pathways [17]. It has been postulated that the bioactive compounds present in PHs can interact with common specific receptors on membranes of the target cells [17]. A model case is the 12 amino acid-long peptides, also known as the “root hair promoting peptide”, which increases root hair development in soybean with a mechanism of action regulated at the gene level [18]. Regardless of the molecular and biochemical mechanism involved, many recent research results support the hypothesis that the biostimulant activity of PHs cannot be explained as a simple result of nitrogen and carbon supplementation [4,14,17,19]. With this regard, it must also be considered that the actual profile of different PHs depends on the protein source and the hydrolysis process, likely resulting in different biostimulant activities [4]. Notwithstanding, the differences across PHs have been poorly studied to date.

On these bases, this work aims to elucidate how different vegetal-derived PHs from different protein sources can modulate plant root development to identify the best biostimulant activity, using tomato as a model crop. In parallel, this work focuses on shedding light on the metabolic processes underlying the differences in rooting observed. To this object, a metabolomics approach was chosen, based on the growing evidence about its potential to unravel metabolic responses to abiotic factors, including biostimulants [9,20].

## 2. Results

### 2.1. Profile of the Protein Hydrolysates and Effects on Tomato Rooting

The different PHs were characterized in terms of C and N content, and then for their phytochemical profile using metabolomics (see Supplementary Material). N ranged from 3.9 to 5.1%, whereas C was in the range 16.9 to 20.0%. PH2, 3, and 4 showed the lowest C/N ratio (3.6), whereas PH6 and 10 had a higher C/N ratio (4.3 and 4.9, respectively) (Supplementary Table S1). Together with annotating several amino acids and their derivatives, metabolomics highlighted a broad and diverse profile of the PHs, that included other N-containing compounds (mainly alkaloids), carbohydrates (mono- to oligosaccharides and reduced sugars), phospholipids-related compounds, fatty acids (with their oxo-derivatives and their coenzyme A thioesters), carotenoids and xanthophylls, phenylpropanoids, steroids, and terpenoids (Supplementary Table S2). The fold-change-based heat map allowed hierarchically clustering the profile of the different PH according to their chemical similarity/dissimilarity (Supplementary Figure S1). In detail, the unsupervised cluster analysis reported as PH2, 3, and 4 clustered together, whereas the other PHs were grouped in a separate sub-cluster.

**Figure 1:**
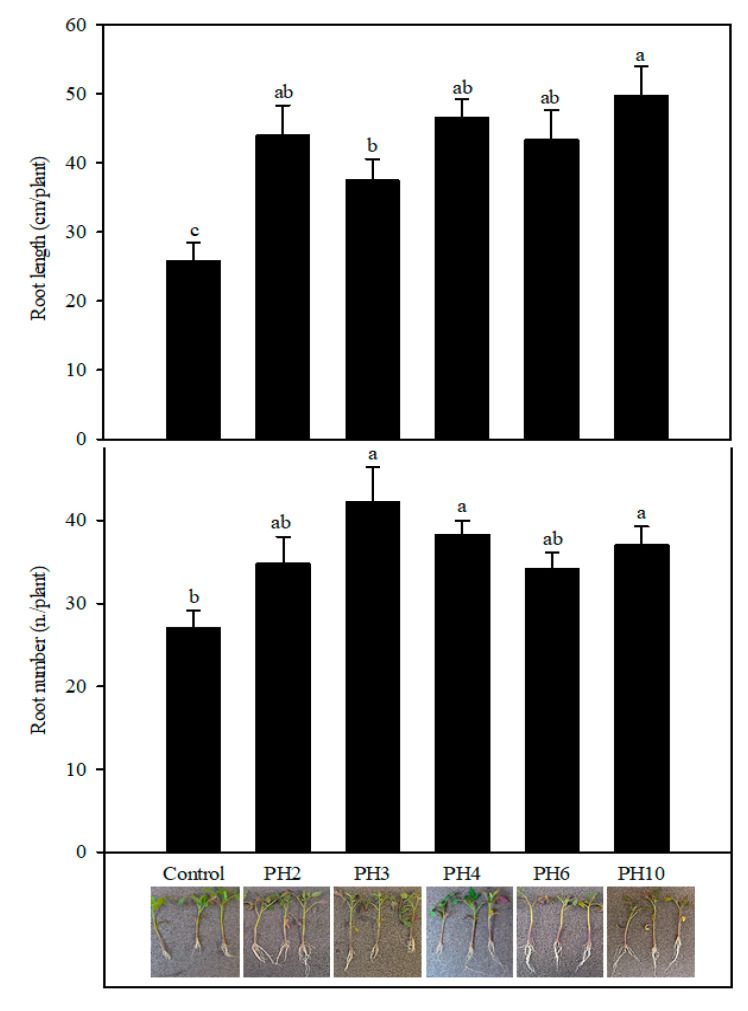
Root length and number of tomato plants as affected by foliar treatment (quick dipping) of tomato cuttings with vegetal-derived protein hydrolysates (PH2, PH3, PH4, PH6, PH10). Vertical bars represent standard error of the means. Different letters indicate different means according to Duncan’s multiple range test (*p* < 0.05).

The rooting test of tomato cuttings was carried out to evaluate the capability of vegetal-derived protein hydrolysates (PHs) to stimulate rooting in tomato cuttings (Figure 1). Root length and number were significantly affected by treatments (*p* < 0.001 and *p* < 0.01, respectively). Root length of PH2, PH3, PH4, PH6, PH10 was significantly higher by 71, 45, 81, 68, and 93% in comparison with control treatment, respectively. Among the tested PHs, root length was higher in PH10 than in PH3 treatment, while the other PHs (PH2, PH4, PH6) gave intermediate values not significantly different from PH10 and PH3. The root number of tomato plants in PH3, PH4, and PH10 was higher by 56, 42, 37% than the control treatment, respectively (Figure 1), while PH2 and PH6 gave intermediate values not significantly different from control and PH3, PH4, and PH10 treatments.

### 2.2. UHPLC/QTOF-MS Metabolic Profiling of Tomato Roots

**Figure 2:**
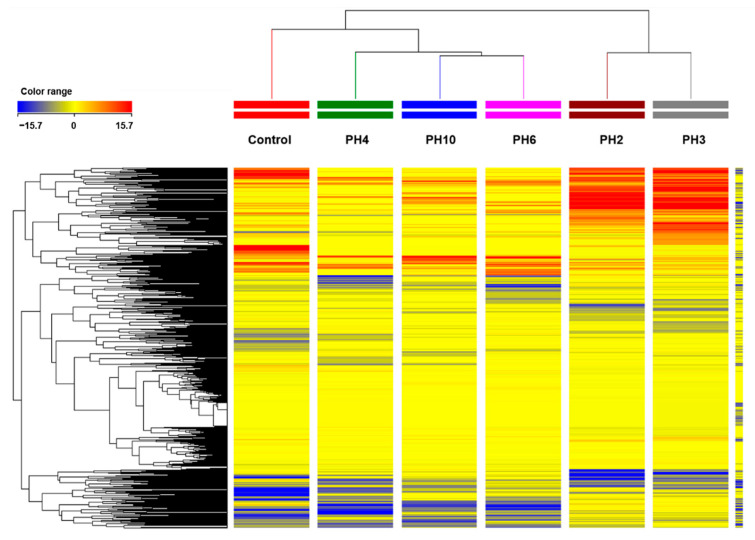
Unsupervised hierarchical cluster analysis carried out from ultra-performance liquid chromatography electrospray ionization quadrupole time-of-flight mass spectrometry (UHPLC-ESI/QTOF-MS) metabolomics analysis of tomato roots after protein hydrolysate (PH) application. The fold-change-based heat map was used to build hierarchical clusters (linkage rule: Ward; distance: Euclidean).

An untargeted metabolomics approach was applied to better understand the effect of PH on plant metabolic processes. Overall, more than 3000 putative compounds were annotated and used for chemometrics and biological interpretation. The whole list of metabolites annotated is provided as Supplementary Material together with their composite mass spectra and individual abundance (Supplementary Table S1). As a preliminary approach, the unsupervised hierarchical cluster analysis was performed to distinguish the metabolic signatures of samples based on the fold-change (Figure 2). The hierarchical cluster analysis (HCA) revealed that metabolomic profiles in roots were significantly influenced by the PHs foliar application, in a PH-dependent specific manner. In fact, samples clustered in two main groups where PH2 and PH3 clustered together while the rest of the PHs clustered independently but closer to the control.

**Figure 3:**
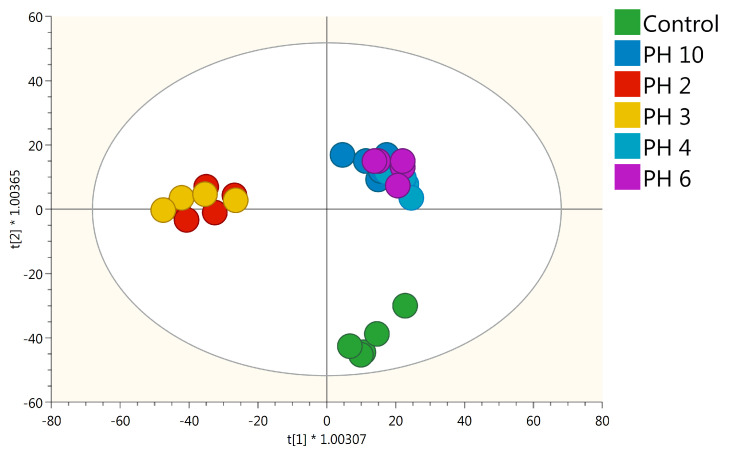
Score plot of orthogonal projection to latent structures discriminant analysis (OPLS-DA) supervised modeling carried out on untargeted metabolomics profiles of tomato roots following PHs application (R^2^Y = 0.89, Q^2^Y = 0.5).

These pieces of evidence were further confirmed by Orthogonal partial least squares discriminant analysis (OPLS-DA), allowing to better separate the samples in the score plot space according to the PH application (Figure 3).

The model was validated by the goodness-of-fit (R2Y > 0.89), the prediction ability (Q2Y > 0.5), and by the cross-validation analysis of variance CV-ANOVA *p*-value < 0.05. In agreement with HCA, all the PH-treated plants were well separated from the control, especially PH2 and PH3 which presented the most distinct metabolic profiles. The VIP analysis was then performed to point out the metabolites explaining the differences observed. Metabolites possessing a VIP score > 1.3 were considered as discriminant. This analysis resulted in 117 compounds that included secondary metabolites such as phenylpropanoids and nitrogen-containing secondary compounds, hormones (i.e., brassinosteroids, gibberellins, cytokinins), and amino acids-related compounds (Supplementary Table S2).

**Figure 4:**
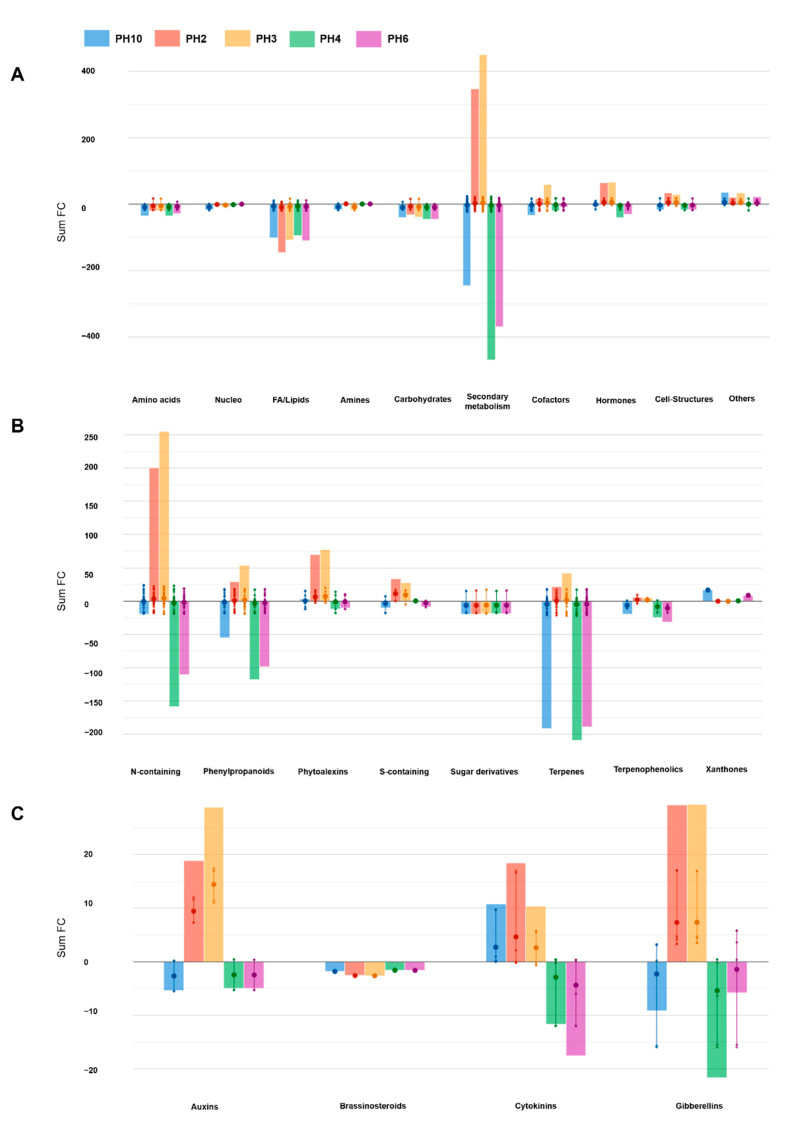
Biosynthetic processes (**A**), secondary metabolism (**B**), and hormone biosynthesis (**C**) processes affected by the PHs. The metabolomic dataset produced through UHPLC-ESI/QTOF-MS was subjected to volcano plot analysis (*p* < 0.01, fold-change > 3) and differential metabolites loaded into the PlantCyc Pathway Tool (https://www.plantcyc.org/ (accessed on 5 January 2021)). The large dot represents the average (mean) of all data values for metabolites and the small dots represents the individual logFC for each metabolite. The *x*-axis represents each set of subcategories while the *y*-axis corresponds to the cumulative fold-change. The abbreviated subcategory names on the *x*-axis correspond to: Nucleo: nucleosides and nucleotides; FA/lipids: fatty acids and lipids; Amines: amines and polyamines; Cofactors: cofactors, prosthetic groups, electron carriers, and vitamins; N-containing: nitrogen-containing secondary metabolism; S-containing: sulfur-containing secondary metabolism.

Thereafter, differential compounds were identified through volcano plot analysis (*p*-value < 0.01; FC > 3). This analysis allowed highlighting 286 metabolites that were then used for interpretation (Supplementary Table S3). Given the broad chemical and biological diversity among the discriminant compounds, a pathway tool analysis from PlantCyc was used to objectively simplify the interpretations (Figure 4). The results of this analysis are summarized in Figure 4. The outcome corroborated the distinctive effect of PH2 and PH3 on root metabolism, compared to the control samples. Notably, half of the significant metabolites belonged to the secondary metabolites. Although the secondary metabolism was strongly modulated by all the PHs, only PH2 and PH3 elicited an accumulation of these metabolites (Figure 4B), in particular, for nitrogen-containing secondary metabolites. PH2 and PH3 strongly elicited alkaloids and glucosinolates, while PH4 and PH6 showed an opposite trend. Similarly, phenylpropanoid and terpenoids were down accumulated following PH10, PH4, and PH6 application. Besides secondary metabolites, fatty acids and, to a lower extent carbohydrate, were down accumulated as a common response to the PHs application. Moreover, the treatments impacted the processes related to cofactors, prosthetic groups, electron carriers, and vitamins. For instance, the tetrapyrrole preuroporphyrinogen was elicited in all cases, while tocopherol was repressed. The plant cell structures hydroxy stearate and hexadecane-diol were up accumulated, while the sinapyl alcohol coniferyl alcohol was down accumulated in the presence of PHs.

Finally, the PH2 and PH3 induced the reprogramming of phytohormones profile by modulating gibberellin and cytokinin biosynthesis and, to a lesser extent, auxins and brassinosteroids biosynthesis (Figure 4C). Particularly, these PHs strongly stimulated the accumulation of the IAA precursors 4-(indol-3-yl) butanoate (IBA) and tryptamine and the gibberellin precursor ent-7α-hydroxykaur-16-en-19-oate. Similarly, several downstream gibberellins were accumulated mainly in the presence of PH2 and PH3. Cytokinins were also stimulated, including zeatin riboside, the conjugate trans-zeatin-O-glucoside-7-N-glucoside, and lupinate. In contrast, a general down accumulation of campest-4-en-3-one and (22R,23R)-28-homocastasterone were observed for all the PHs (Table 1).

## 3. Discussion

Protein hydrolysates have extensively demonstrated their ability to promote plant growth and development, positively regulating plant biomass and crop performance, even under environmental constraints [14]. Despite the number of scientific works focused on unravelling the mechanism of action elicited by the application of PHs, the underlying metabolic response is still poorly understood. So far, several mechanisms at the basis of the response to PHs have been proposed, like the stimulation of enzymes related to N metabolism, the establishment of hormone-like activities, as well as an improved micro and macro nutrient-acquisition [4,7,19].

In our work, we illustrate how foliar application of five different vegetal-derived PHs significantly enhanced rooting in tomato cuttings, along with eliciting a deep reprogramming at the metabolome level. Both unsupervised clustering and the supervised OPLS-DA multivariate modeling indicated that all PHs-treated plants have distinct metabolome profiles compared to control (Figure 3). Regarding the VIP analysis, we evidenced that both primary and secondary metabolism, the latter to a major extent, were modulated by PHs-treatment. Two main clusters could be distinguished (i.e., PH2 and PH3 vs. PH4, PH6, and PH10) (Figure 2), characterized by opposite accumulation trends of the same classes of molecules, with special regard to secondary metabolites (Figure 4). Moreover, a general impairment in fatty acid, lipids, and carbohydrate compounds resulted in a common response to different PH-treatments. In particular, in lipid metabolism, the main imbalance was related to phosphatidylcholines, where PH2 and PH3 showed a strong down-accumulation on the contrary of PH4, PH6, and PH10. Sterols and glycolipids down accumulated in response to all PHs. Alteration of fatty acid and lipid metabolism can be the consequence of either the remodeling of cell membranes, which naturally occurs during root development or being part of a signal transduction cascade [21,22]. Dynamics in lipids profiles of cellular membranes determine their physical properties, like fluidity and permeability, which influence plant response to diverse environmental stimuli [23,24].

All PHs significantly enhanced root length in comparison with control treatment, while root number was significantly increased only by PH3, PH4, and PH10. Our findings agree with previous works that reported root morphological changes induced by PHs, in terms of increase of both number and length of adventitious roots of tomato cuttings [7,19]. The above findings demonstrated a hormone-like activity of PH, corroborating our results about the shaping of phytohormones profile we observed.

Root development is regulated by coordinated crosstalk between the major phytohormones (auxins, cytokinins, gibberellins), of which auxin is the strongest growth-promoting effector [25,26]. Endogenous indole-3-acetic acid (IAA) is mostly synthesized in meristematic tissues, primarily in young and not fully expanded leaves, and then transported through the polar auxin transport (PAT) to root tips, where auxin accumulates and performs its role [27]. The IAA gradient needed for root development is also maintained by local auxin biosynthesis, conjugation, degradation, and storage, which can occur in any part of the root system [28]. Cytokinins act as an antagonist to auxin; thus, the fine regulation of the auxin/cytokinin ratio influences the trade-off between mitotic activity and cell differentiation, determining root development [29,30]. The roles of gibberellins and brassinosteroids are less understood, but their involvement is broadly demonstrated [7,31]. Brassinosteroids act as antagonist to auxin to control the spatiotemporal balance of stem cell dynamics in root tips [26,32].

Interestingly, all this complex crosstalk of signals relates to our results, supporting a hormone-like activity in response to PHs, in accordance with the morphological changes measured. Indeed, all PHs stimulated changes in hormone profiles relating to root development. Auxins and gibberellins accumulated in response to PH2 and PH3 treatment, and down accumulated in PH4, PH6, and PH10-treated plants. In respect of auxin-mediated root development, the general accumulation of gibberellins can stimulate PAT by up-regulating the key auxin transporter PIN1 and thus, promoted cell proliferation following PH2 and PH3 treatments. Notably, these two PHs determined the strong accumulation of 4-(indol-3-yl) butanoate (IBA) and tryptamine, both endogenous precursor of IAA [33]. IBA, as a positive regulator of root-growth, plays an important role after its reversible conversion to IAA by peroxisomal enzymes, as well as a signaling molecule, beyond that of being a simple cell-storage for IAA [33]. Cytokinins are positive regulators of cell division and root elongation [34,35]. Among all cytokinins, we detected an increase in zeatin level in response to PH2, and with a lesser extent, to PH3 and PH10. Guan and colleagues [36] proposed zeatin as a positive regulator of root extension and negative regulator of root initiation in tomato cuttings, similarly to cytokinin function during lateral root development [37,38]. The complex interplay between cytokinins and auxins may have played a pivotal role in root morphogenesis and architecture of tomato cutting in our experiments. Nonetheless, a general down accumulation of brassinosteroids was also observed following all PH treatments. The catabolism of these phytohormones is critical in the maintenance of the homeostasis of endogenous brassinosteroids, which act antagonistically to auxins in regulating root cell elongation [39].

Our results report the metabolic and morphological responses in tomato root tissues at 7 days after PH-treatment. At this time-point, it can be postulated that the auxin/cytokinins ratio was shifting in favor of cytokinins in roots of tomato plants treated with PH2, PH3, and PH10, since they started to accumulate root-synthesized cytokinins. Moreover, the higher amount of IBA detected in roots treated with PH2 and PH3 promoted a longer-lasting auxin response. It can be speculated that these two hormones might prompt the root architecture differently in the later days, beyond what was visible at 7-days after wounding.

Considering secondary metabolism, the major differences were visible in the profiles of N- and S-containing compounds, as well as phenylpropanoids. Nitrogen-containing compounds accumulated, especially in response to PH2 and PH3 treatments, possibly reflecting a PH-induced enhancement of nitrogen uptake, as previously reported [14]. Moreover, it must be considered that these two PHs were characterized by a low C:N ratio, thus being relatively higher in N-containing compounds (Supplementary Table S1). Among others, glucosinolate (GSLs) compounds were strongly modulated by the treatments, mainly concerning aliphatic and, to a lower extent, indole GSLs. GSLs are nitrogen and sulfur containing-molecules, typically associated with biotic attacks but also involved in response to abiotic stresses such as nutritional, drought, and light stress [40]. Recent works are opening new perspectives about GSLs involvement in root development and gravitropism [41]. The modulation depicted in nitrogen metabolism also altered alkaloid levels, since their biosynthesis is directly connected to nitrogen-containing precursors [42]. Indeed, alkaloids are important nitrogen storage for plants, especially under nutrient deficiencies, and their accumulation correlates with nitrogen availability and origin [43]. Increased levels of alkaloids can establish tolerance against environmental constraints, behaving as protective agents, primarily inhibiting oxidative stress, but also regulating plant growth [43,44].

From a general perspective, it is still a matter of debate whether the biostimulant effect of PHs can relate to their ability to trigger and sustain developmental processes via signaling mechanisms. In our work, we demonstrated that the quick dipping of tomato cuttings into PH can affect the metabolism and the morphology of distant organs like roots. In this respect, the diverse modulation at metabolome level in response to different PHs is probably linked to the heterogeneous origins of the five vegetal-derived PHs tested, and thus to the different composition of the PHs. PHs are mainly composed of peptides, amino acids, and other plant macromolecules and the putative signal cascade can be promoted by small peptides. On the other hand, an exemplary case of a small peptide regulating root growth is the dodecapeptide RHPP [18]. Indeed, other recent works are opening new perspectives on the role of small peptides as a novel class of phytohormones, orchestrating plant development and growth via cell-to-cell communication [45]. A very recent publication demonstrated the importance of some biostimulant molecular size fractions to unravel the role of vegetal-derived PHs on the metabolic and morphological modulation of root development [19]. This previous work found that the PH fraction containing low molecular weight compounds (MW < 1 kDa) like small oligopeptides (i.e., 7–8 amino acids) was the most bioactive fraction. Noteworthy, such the smallest fraction of a legume-derived PH was associated with auxin-like and root-promoting activities [19]. Herein, we are providing evidence that such hormone-like activity of PHs also depends on the protein source. On the other hand, the product-specific modulation of plant metabolism suggests that generalized outcomes are not appropriate and paves the way towards well-defined claims for specific products.

## 4. Materials and Methods

### 4.1. Selection and Characterization of Protein Hydrolysates

Five PHs were used in the experiment. One of them (PH4) was a commercial product resulting from enzymatic hydrolysis of legume-derived proteins. The other four PHs were made by enzymatic hydrolysis of other vegetal-derived proteins as described previously [46]. Briefly, dry biomass from different plant sources belonging to Fabaceae (PH10), Malvaceae (PH3), Brassicaceae (PH6), and Solanaceae (PH2) were ground into powder; the powdered biomass was mixed in water, homogenized at a speed of 8000× *g* for 3 min and extracted by stirring for 6 h at 50 °C. The water suspension was filtered and centrifuged (8000× *g* for 60 min) to separate proteins, and the enzymatic hydrolysis of proteins was performed using trypsin (enzyme-substrate ratio of 1:100 [*v/v*]) at 37 °C for 3 h. The enzyme was inactivated by heating at 100 °C for 5 min. The resulting hydrolysates were then rapidly cooled to ambient temperature in the ice bath. Soluble compounds like amino acids and peptides were removed from the insoluble compounds by centrifugation (10,000× *g* for 30 min), and the resulting solution concentrated 6 times in a rotary evaporator under vacuum at 60 °C.

Total nitrogen and carbon were quantified through the Dumas’ method using an elemental analyzer (Elemental vario MAX CN, Langenselbold, Germany). Thereafter, the different PH were characterized through liquid chromatography quadrupole-time-of-flight mass spectrometry (UHPLC/QTOF-MS) for their metabolite profile.

### 4.2. Growth Conditions, Plant Material, and In Vivo Assay

Tomato (*Solanum lycopersicum* L. cv. Akrai F1, SAIS Sementi, Cesena, Italy) seeds were surface sterilized with 2% sodium hypochlorite for 20 min, then washed with distilled water and sown in a tray filled with a commercial peat moss-based substrate (Brill, Gebr. Brill Substrate GmbH & Co., Georgsdorf, Germany), in a growth chamber at Tuscia University, Italy. In the growth chamber, the photoperiod was 12 h, light intensity was set to 450 μmol m^−2^ s^−1^, temperature was kept at 24 °C, and the relative humidity constantly maintained at 70%. At 15 days after sowing, the tomato seedlings, at three-true-leaf stage, were cut to 1 cm from the plants’ collar. The cuttings were dipped into a water solution of different PHs at 8 g L^−1^, using a quick dip method of the leaves for 3 s [7]. The unrooted cuttings were then placed in transparent polypropylene microboxes with filtered covers (diameter 90 mm, height 140 mm) containing a layer of 70 mm of wetted quartziferous sand. The microboxes were closed with parafilm to achieve a relative humidity close to saturation (100%). Treatments were arranged in a completely randomized block design with three replicates; each experimental unit consisted of a microbox containing 5 cuttings. At 7 days from planting, the roots were harvested and gently rinsed with distilled water. For root morphology determination, 10 cuttings per each treatment were selected. Roots were separated from the stems using a surgical blade and the number of the adventitious roots were counted manually. Entire root systems were scanned using an Epson Perfection V700 Photo scanner; the scanned images were used to determine total root length and number using WinRHIZO (Regent Instrument Inc., Quebec, QC, Canada) [47].

### 4.3. UHPLC/QTOF-MS Untargeted Metabolomics

Roots from five cuttings per treatments were analyzed by untargeted metabolomics according to a previously reported approach [48]. Briefly, root samples were extracted by using a homogenizer-assisted extraction in 80% methanol solution with 0.1% (*v*/*v*) formic acid, centrifuged and filtered through 0.22 µm cellulose filters. Metabolomics analysis was then carried out through UHPLC/QTOF-MS. A water-acetonitrile reverse phase gradient elution (6% to 94% acetonitrile in 34 min) and positive polarity SCAN acquisition (range 100–1200 *m*/*z*) were used for chromatography and electrospray mass spectrometry, respectively, as previously set up [49]. The injection sequence was randomized, and blank samples (extraction solvent only) injected at the beginning and at the end of the sequence. Besides, quality control samples (QCs) were randomly analyzed throughout the sequence, using the same chromatographic method but in a data-dependent tandem MS/MS mode (10 precursors per cycle, 1 Hz, 50–1200 *m*/*z*), at different collision energies (10, 20, and 40 eV) [50]. QCs were used to increase confidence in the annotation.

The raw mass features were processed according to a targeted ‘find-by-formula’ algorithm by the Agilent Profinder B.06 (Agilent Technologies, Santa Clara, CA, USA) software. In particular, the isotopic pattern (monoisotopic mass and isotopes profile), adopting a mass tolerance of 5-ppm, was applied following mass and retention time alignment. For annotation purposes, the comprehensive database PlantCyc 9.6 (Plant Metabolic Network, http://www.plantcyc.org (accessed on 5 January 2021)) was used. Therefore, in our untargeted conditions, a Level 2 of annotation (i.e., putatively annotated compounds) was achieved, as reported by COSMOS Metabolomics Standards Initiative [51]. The compounds annotation step was strengthened by processing QCs in MS-DIAL 4.24 [52]. To this aim, both the MS/MS experimental spectra available in MS-DIAL (MONA-Mass Bank of North America) and the in-silico fragmentation spectra produced through MS-Finder from the compounds in PlantCyc [53] were used.

### 4.4. Statistical Analysis

Root length and number in tomato plants were subjected to ANOVA test using the software package SPSS 10 for Windows (SAS Inc., Cary, NC, USA). Duncan’s multiple range test was performed at *p* = 0.05 on each of the significant variables measured.

Metabolomics data were analyzed through Agilent Technologies Mass Profiler Professional 12.6, where abundance was log2 transformed, normalized, and baselined against the median [50]. At first, unsupervised hierarchical cluster analysis (Euclidean distance, Ward’s linkage) was used to describe similarity/dissimilarity across treatments. Thereafter, the dataset was exported into Simca+ (Umetrics, Malmo, Sweden) for the supervised modeling by orthogonal projection to latent structures discriminant analysis (OPLS-DA). Therein, the model goodness parameters, namely correlations R2X, R2Y, and Q2Y prediction ability, were calculated. In particular, we retained those metabolites presenting a VIP score (variable importance in projection) > 1. A volcano plot analysis was finally done by combining ANOVA (*p* < 0.05; Bonferroni multiple testing correction) and fold-change analysis (cut-off ≥ 2), and differential compounds interpreted using the PlantCyc Pathway Tool [54,55].

## 5. Conclusions

The quick dipping of tomato cuttings into diluted solutions of protein hydrolysates significantly boosted the plant rooting process. Noteworthy, evidence at the molecular level in roots supported the biostimulant effect we observed. Noteworthy, it is important to highlight that biostimulant activity goes beyond plant nutrition processes, and that protein hydrolysates from different botanical origins provided distinctive responses during the rooting process. The ability of protein hydrolysates to interfere with plant signaling processes has been confirmed to be a pivotal process for the biostimulant activity. Nonetheless, it is remarkable that several secondary metabolites differentially accumulating in root tissues in the treatments paved the way towards an improved resilience against plant stress conditions. With this regard, further work should be devoted to providing more information regarding the ability of the plant biostimulant to boost crop performances in response to environmental challenges.

## Figures and Tables

**Table 1:** Differential metabolites included in the biosynthetic pathways related to hormones, as classified by the PlantCyc Pathway Tool (https://www.plantcyc.org/ (accessed on 5 January 2021)).

		*Log FC*				
*Class*	Compound	PH10	PH2	PH3	PH4	PH6
**Auxin Biosynthesis**	4-(Indol-3-Yl)Butanoate	0.16	7.25	17.46	0.4	0.35
	Tryptamine	−5.53	11.61	11.44	−5.34	−5.34
**Brassinosteroid Biosynthesis**	Campest-4-En-3-One	−1.85	−2.62	−2.64	−1.62	−1.65
**Cytokinin Biosynthesis**	*cis*-Zeatin Riboside	9.72	2.1	5.74	−12.04	−12.07
	Lupinate	0.93	−0.21	−0.17	−0.29	−6.05
	*trans*-Zeatin-O-Glucoside-7-N-Glucoside	−0.03	16.62	−0.67	0.23	0.16
	N^6^-(Δ^2^-Isopentenyl)-Adenosine 5′-Monophosphate	0.16	−0.02	5.48	0.4	0.35
**Gibberellin and Gibberellin Precursor Biosynthesis**	Gibberellin A_38_	3.14	3.3	3.48	−6.42	3.6
	Gibberellin A_25_	0.16	17.12	16.92	0.4	0.35
	Gibberellin A_9_	3.21	4.2	4.65	−0.19	5.79
	*ent*-7α-Hydroxykaur-16-En-19-Oate	−15.75	4.66	4.36	−15.51	−15.56
**Abscisic Acid Degradation**	Β-D-Glucopyranosyl abscisate	−1.83	−2.4	−2.22	−1.65	−1.98
	Dihydroxyphaseic Acid	−0.56	−5.47	−12.33	−9.87	−16.96
**Cytokinin Degradation**	Isopentenyl Adenosine	−14.43	−0.15	−0.36	−17.64	−10.93
	*trans*-Zeatin Riboside	22.16	3.61	3.38	0.39	0.37
**Gibberellin Degradation**	Gibberellin A_9_	3.21	4.2	4.65	−0.19	5.79
	16α,17-Epoxy Gibberellin A_4_	−3.32	13.25	14.95	−3.08	−3.13

## Data Availability

The datasets generated for this study are available as Supplementary Materials.

## Supplementary Materials

The following are available online at https://www.mdpi.com/2223-7747/10/2/326/s1, Table S1. Whole dataset produced from untargeted metabolomics carried out in tomato roots after PHs application. Compounds are presented with individual intensities and with composite mass spectra (monoisotopic accurate mass/abundance combinations); Table S2. Discriminant metabolites identified by the VIP analysis in tomato roots after PHs application. Compounds were selected from OPLS-DA multivariate modelling as discriminant when having a VIP score > 1.3; Table S3. Differential metabolites derived from Volcano analysis (*p*-value < 0.01, Bonferroni multiple testing correction; fold-change threshold FC > 3) in tomato roots metabolomic profiles following PHs application. Table S1. Carbon and nitrogen content, together with C/N ratio, of the different protein hydrolysates tested. Table S2. Untargeted metabolite profile in the different protein hydrolysates tested; individual abundances are provided for each test material. Figure S1. Hierarchical cluster analysis carried out from the chemical profile of the different protein hydrolysates; a fold-change heat map was made, and Euclidean distance used for clustering.
