# Supplementary material for: Foliar Application of Different Vegetal-Derived Protein Hydrolysates Distinctively Modulates Tomato Root Development and Metabolism
Source: Plants (Basel). 2021 Feb 8;10(2):326. doi: 10.3390/plants10020326 (PMC7914860; doi:10.3390/plants10020326)
Supplement: Supplementary file 1 [file plants-10-00326-s001.zip › plants-1091060 - supplementary proof done.pdf]

Table S1. C and N content in the different protein hydrolysates (PHs).

Protein hydrolysate	C (%)	N (%)	C/N
PH2	18.6	5.2	3.6
PH3	16.9	4.7	3.6
PH4	17.6	4.9	3.6
PH6	17.1	3.9	4.3
PH10	20.0	4.9	4.1

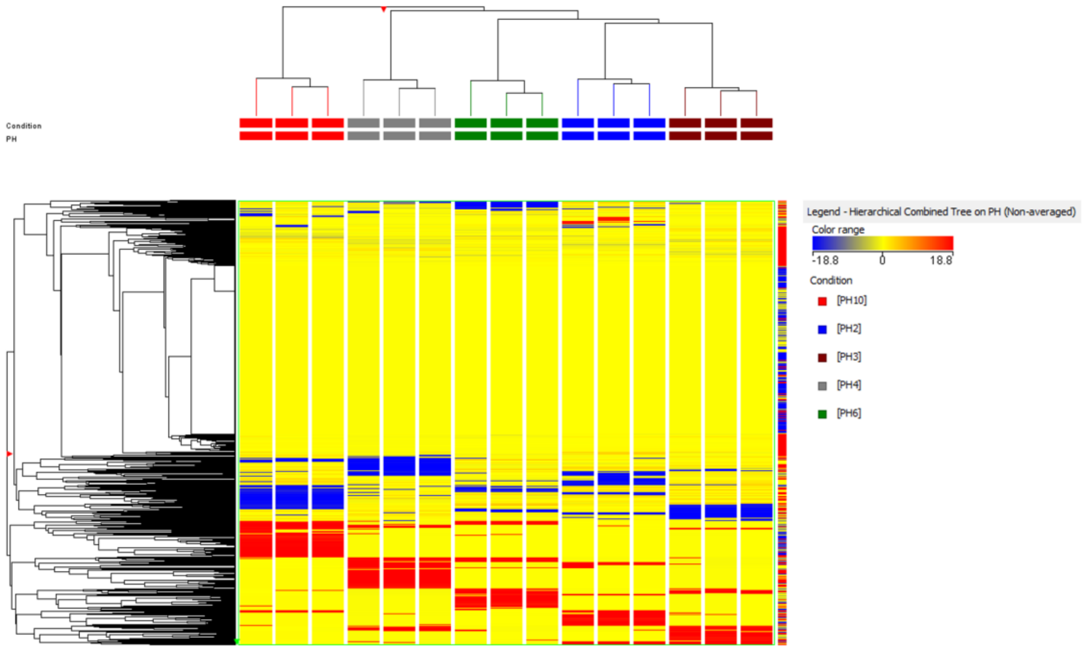

Figure S1. Hierarchical cluster analysis carried out from the chemical profile of the different protein hydrolysates; a fold-change heat map was made, and Euclidean distance used for clustering.
